# *MICA* diversity and linkage disequilibrium with *HLA-B* alleles in renal-transplant candidates in southern Brazil

**DOI:** 10.1371/journal.pone.0176072

**Published:** 2017-04-18

**Authors:** Roger Haruki Yamakawa, Patrícia Keiko Saito, Geórgia Fernanda Gelmini, José Samuel da Silva, Maria da Graça Bicalho, Sueli Donizete Borelli

**Affiliations:** 1 Department of Basic Health Science, Universidade Estadual de Maringá – Maringá, Paraná, Brazil; 2 Laboratório de Imunogenética e Histocompatibilidade, Department of Genetics, Universidade Federal do Paraná – Curitiba, Paraná, Brazil; Morehouse School of Medicine, UNITED STATES

## Abstract

The major histocompatibility complex (MHC) class I chain-related gene A (*MICA*) is located centromerically to the human leukocyte antigen (*HLA)-B*. The short distance between these loci in the MHC indicates the presence of linkage disequilibrium (LD). Similarly to the *HLA*, the *MICA* is highly polymorphic, and this polymorphism has not been well documented in different populations. In this study, we estimated the allelic frequencies of *MICA* and the linkage disequilibrium with *HLA-B* alleles in 346 renal-transplant candidates in southern Brazil. *MICA* and *HLA* were typed using the polymerase chain reaction-sequence-specific primer method (PCR-SSO), combined with the Luminex technology. A total of 19 *MICA* allele groups were identified. The most frequent allele groups were MICA*008 (21.6%), MICA*002 (17.0%) and MICA*004 (14.8%). The most common haplotypes were MICA*009-B*51 (7.8%), MICA*004-B*44 (6.06%) and MICA*002-B*35 (5.63%). As expected from the proximity of the *MICA* and *HLA-B* loci, most haplotypes showed strong LD. Renal patients and healthy subjects in the same region of Brazil showed statistically significant differences in their *MICA* polymorphisms. The MICA*027 allele group was more frequent in renal patients (Pc = 0.018, OR: 3.421, 95% CI: 1.516–7.722), while the MICA*019 allele group was more frequent in healthy subjects (Pc = 0.001, OR: 0.027, 95% CI: 0.002–0.469). This study provided information on the distribution of *MICA* polymorphisms and linkage disequilibrium with *HLA-B* alleles in Brazilian renal-transplant candidates. This information should help to determine the mechanisms of susceptibility to different diseases in patients with chronic kidney disease, and to elucidate the mechanisms involved in allograft rejection associated with *MICA* polymorphisms in a Brazilian population.

## Introduction

The major histocompatibility complex (MHC) class I chain-related gene A (*MICA*) is one of the highly polymorphic genes located in the human MHC [1–3] and is located 46 kb centromerically from the human leukocyte antigen *(HLA)-B*. The short distance separating *MICA* from *HLA*-*B* in the MHC indicates the presence of linkage disequilibrium between these loci [1, 2, 4].

Several studies have demonstrated the role of *MICA* polymorphism in a large number of diseases, and the immune response against *MICA* antigens may correlate with acute and chronic rejection of various organs, including renal transplants [5–9]. Similarly to the known involvement of preformed antibodies against the *HLA* antigens in acute and chronic rejection of a graft [8, 10–12], many studies have shown the importance of MICA alloantibodies in the rejection of various organs [13, 14].

The Brazilian population is one of the most ethnically diverse in the world [15], which may impede the search for a matching, unrelated donor. Previous studies have reported some ethnic differences in the distribution of *MICA* polymorphisms, similar to those found for *HLA* polymorphisms [16–18]. However, *MICA* polymorphisms in different populations have not been as well documented as those of *HLA*. To our knowledge, no studies in Brazil have investigated the *MICA* allelic diversity and linkage disequilibrium with *HLA-B* in renal-transplant candidates.

To fill this gap, we evaluated the *MICA* diversity and linkage disequilibrium with *HLA-B* alleles in renal-transplant candidates in a population in southern Brazil.

## Materials and methods

### Samples

The study included a total of 346 patients (female/male: 135/211) with chronic kidney disease who were renal-transplant candidates and were registered at two regional transplant centers, the Central Regional de Transplantes Norte/Londrina (CRTN/Londrina) and the Central Regional de Transplantes Noroeste/Maringá (CRTNO/Maringá), in northern and northeastern Paraná, respectively, in the period from July 2010 to March 2011. Inclusion criteria were patients with current data (active patients and potential recipients), on dialysis for at least 60 days and to give informed consent to participate in the study. Age over 18 years was considered as exclusion criteria. The study was approved by the Ethics Committee of the Universidade Estadual de Maringá (Protocol No. 333/2011). All procedures followed Resolution 196/1996 of the Brazilian Health Council, which rules on research involving humans. All procedures were explained to each subject, and written informed consent was obtained from each subject.

### DNA extraction and HLA-B and MICA typing

To perform the *HLA-B* and *MICA* typing, about 5 mL of blood was collected by venipuncture in vacuum tubes (Vacutainer, Becton and Dickson, Oxford, UK) containing ethylene diamine tetraacetic acid (EDTA) as anticoagulant. Then, we extracted the genomic DNA by the separation-column method, using the Biopur kit for DNA extraction (Biometrix, Curitiba, Paraná, Brazil), following the manufacturer's protocol. After adjusting the DNA concentration, obtained by the optical-density method, we amplified the DNA using polymerase chain reaction-sequence specific primers (PCR-SSO) combined with Luminex technology. The genomic DNA was amplified using biotinylated sequence-specific primers for *HLA-B* and *MICA* in a GeneAmp PCR System 9700 thermal cycler (Applied Biosystems, Foster City, CA, USA), followed by hybridization with complementary probes for DNA, conjugated with microspheres (beads) labeled with different fluorochromes to identify complementary sequences of the amplified DNA, using the LABType kit (One Lambda, Inc., Canoga Park, CA, USA), following the manufacturer's protocol. After hybridization, the results were read using the flow cytometry platform LABScan^™^100 (One Lambda, Inc.), followed by analysis using the program HLA Fusion version 2.0 (One Lambda, Inc.). The results showed low-medium resolution.

### Comparison of the results with published data

In the *HLA-B* and *MICA* comparisons, this study used as control the data published by Ribas et al. (2008)[17], since their study was carried out in the same region of Brazil.

### Statistical analyses

The Arlequin software package version 3.11 [19] was used to calculate the allele and haplotype frequencies and to assess the Hardy-Weinberg equilibrium. The haplotype frequencies were estimated using the expectation-maximization algorithm (maximum-likelihood method) as included in Arlequin 3.11. The values for relative linkage disequilibrium (LD) between pairs of *MICA* and *HLA-B* allele groups and their level of significance (p values) were determined with the same software package. The overall comparison of *HLA* and *MICA* allelic frequencies between renal patients and healthy subjects [17] was performed with a G-test, and individual comparisons were performed using Fisher's exact test. Statistically significant differences (P≤0.05) were corrected by the Bonferroni method for multiple comparisons (Pc). Odds ratios (OR) and 95% confidence intervals (CI) were calculated.

## Results

The *MICA* and *HLA-*B allelic frequencies are shown in Tables 1 and 2, respectively. A total of 29 *HLA-*B and 19 *MICA* allele groups were identified. The most common allele groups were HLA-B*35 (10.5%), HLA-B*44 (9.9%), HLA-B*51 (9.6%), MICA*008 (21.6%), MICA*002 (17.0%) and MICA*004 (14.8%).

**Table 1:** Allele group frequencies of *MICA* in all samples (n = 346), and comparison with healthy subjects.

*MICA*	Renal	%	Healthy subjects (Ribas et al., 2008)[17]	%	*p*	*p*_*c*_	*OR* (95% CI)
*001	9	1.30%	4	0.98%	0.436	1	
*002	118	17.05%	72	17.65%	0.437	1	
*004	103	14.88%	47	11.52%	0.068	1	
*006	4	0.58%	3	0.74%	0.480	1	
*007	17	2.46%	13	3.19%	0.385	1	
*008	150	21.68%	108	26.47%	0.047	1	
*009	95	13.73%	53	12.99%	0.401	1	
*010	46	6.65%	29	7.11%	0.469	1	
*011	21	3.03%	19	4.66%	0.122	1	
*012	9	1.30%	4	0.98%	0.436	1	
*015	7	1.01%	3	0.74%	0.457	1	
*016	23	3.32%	7	1.72%	0.079	1	
*017	14	2.02%	7	1.72%	0.455	1	
*018	32	4.62%	16	3.92%	0.349	1	
*019	0	0.00%	10	2.45%	0.000	0.001	0.027 (0.002–0.469)
*021	0	0.00%	1	0.25%	0.371	1	
*027	39	5.64%	7	1.72%	0.001	0.018	3.421 (1.516–7.722)
*044	1	0.14%	0	0.00%	0.629	1	
*045	1	0.14%	0	0.00%	0.629	1	
*046	1	0.14%	0	0.00%	0.629	1	
*049	0	0.00%	3	0.74%	0.051	1	
*052	2	0.29%	0	0.00%	0.396	1	

P = P-value; Pc = P-value adjusted for multiple comparisons; OR: odds ratio; CI: confidence interval.

Healthy subjects described by Ribas et al., (2008) [17].

**Table 2:** Allele group frequencies of *HLA-B* in all samples (n = 346), and comparison with healthy subjects.

*HLA-B*	Renal	%	Healthy subjects (Ribas et al., 2008)[17]	%
*07	45	6.50%	28	6.86%
*08	41	5.92%	27	6.62%
*13	11	1.59%	9	2.21%
*14	22	3.18%	26	6.37%
*15	59	8.53%	45	11.03%
*18	34	4.91%	21	5.15%
*27	21	3.03%	11	2.70%
*35	73	10.55%	40	9.80%
*37	9	1.30%	4	0.98%
*38	23	3.32%	10	2.45%
*39	24	3.47%	18	4.41%
*40	36	5.20%	13	3.19%
*41	13	1.88%	6	1.47%
*42	15	2.17%	5	1.23%
*44	69	9.97%	46	11.27%
*45	14	2.02%	6	1.47%
*46	1	0.14%	0	0.00%
*47	1	0.14%	1	0.25%
*48	3	0.43%	2	0.49%
*49	22	3.18%	9	2.21%
*50	17	2.46%	6	1.47%
*51	67	9.68%	42	10.29%
*52	14	2.02%	8	1.96%
*53	15	2.17%	4	0.98%
*54	2	0.29%	0	0.00%
*55	7	1.01%	3	0.74%
*56	1	0.14%	2	0.49%
*57	18	2.60%	7	1.72%
*58	15	2.17%	9	2.21%

Healthy subjects described by Ribas et al., (2008)[17].

The *MICA* allele distribution was in Hardy-Weinberg equilibrium (p>0.05), the observed heterozygosity was 82.0% and the expected heterozygosity was 87.9%. In contrast, the observed and expected heterozygosity of the *HLA-B* allelic distribution differed significantly (p = 0.009): the observed heterozygosity was 90.7% and the expected, 94.1%.

The overall comparison of *MICA* allele frequencies between renal patients and healthy subjects indicated a significant difference between the groups (p<0.0001). In individual comparisons, the MICA*027 allele was more frequent in renal patients (Pc = 0.018, OR: 3.421, 95% CI: 1.516–7.722), while the MICA*019 allele was more frequent in the healthy population (Pc = 0.001, OR: 0.027, 95% CI: 0.002–0.469). The MICA*008 allele was more frequent in healthy subjects; however, with the Bonferroni correction, no statistically significant difference was apparent.

The *HLA-B* allele frequencies did not differ significantly between renal patients and healthy subjects.

The result for haplotype inference showed a total of 77 haplotypes, of which 23 had a frequency greater than 1%. Table 3 shows the frequencies and linkage disequilibrium (LD) values for all haplotypes with a frequency greater than 1% in both studies. The supplementary table (S1 Table) presents a graphical view of the LD parameters and frequencies for all haplotypes characterized in our study.

**Table 3:** *MICA–HLA-B* haplotype frequencies and relative LD values (D’) for haplotypes with a frequency exceeding 1% in all samples (n = 346), and comparison with healthy subjects.

*Haplotype*	Renal				Healthy subjects (Ribas et al., 2008)[17]			
	n	%	D'	p LD	n	%	D'	p LD
MICA*009-B*51	54	7.80%	0.775	0	33	8.09%	0.75	0
MICA*004-B*44	42	6.07%	0.540	0	24	5.88%	0.47	0
MICA*002-B*35	39	5.64%	0.439	0	25	6.13%	0.54	0
MICA*008-B*07	38	5.49%	0.801	0	25	6.13%	0.86	0
MICA*008-B*08	36	5.20%	0.844	0	24	5.88%	0.85	0
MICA*010-B*15	36	5.20%	0.762	0	24	5.88%	0.81	0
MICA*018-B*18	26	3.76%	0.803	0	15	3.68%	0.93	0
MICA*002-B*39	24	3.47%	1.000	0	18	4.41%	1.00	0
MICA*002-B*38	23	3.32%	1.000	0	10	2.45%	1.00	0
MICA*008-B*44	22	3.18%	0.130	0.0301	20	4.90%	0.26	0.0017
MICA*004-B*49	21	3.03%	0.947	0	9	2.21%	1.00	0
MICA*016-B*35	21	3.03%	0.903	0	7	1.72%	1.00	0
MICA*011-B*14	20	2.89%	0.951	0	19	4.66%	1.00	0
MICA*008-B*15	18	2.60%	0.113	0.0852	9	2.21%	20.12	0.6207
MICA*007-B*27	16	2.31%	0.939	0	11	2.70%	1.00	0
MICA*004-B*42	15	2.17%	1.000	0	5	1.23%	1.00	0
MICA*002-B*58	14	2.02%	0.920	0	9	2.21%	1.00	0
MICA*017-B*57	13	1.88%	0.927	0	7	1.72%	1.00	0
MICA*004-B*41	12	1.73%	0.910	0	6	1.47%	1.00	0
MICA*009-B*50	12	1.73%	0.659	0	5	1.23%	0.81	0
MICA*008-B*40	11	1.59%	0.113	0.1842	7	1.72%	0.38	0.0158
MICA*009-B*35	10	1.45%	-0.002	0.9938	6	1.47%	0.01	0.8052
MICA*008-B*13	9	1.30%	0.768	0	8	1.96%	0.85	0
Other**	160	23.12%			82	20.10%		

**Haplotypes with frequency below 1%;

D’ = Relative linkage disequilibrium value; Only haplotypes in attraction (D' = 1 and D'<1) are shown. Healthy subjects described by Ribas et al., (2008)[17];

The most frequent haplotype was MICA*009-B*51 (7.8%), followed by MICA*004-B*44 (6.0%) and MICA*002-B*35 (5.6%). The analysis of linkage disequilibrium (LD) showed that 8 haplotypes had a relative LD value (D’) of 1.

No statistically significant difference was observed in the frequency of those *MICA–HLA-B* haplotypes with a frequency greater than 1%, in both studies.

## Discussion

The vast majority of studies have addressed the *MICA* polymorphism of an entire population, using apparently healthy subjects [16–18, 20].

The Brazilian population has wide genetic heterogeneity, composed of a mix of ethnic groups, a result of immigration from several countries during the colonization of Brazil, resulting in an interracial mixture of Europeans, Africans, Amerindians and Asians [15]. The samples evaluated in this study are from the north/northeastern region of Paraná, in southern Brazil. The southern region, including the state of Paraná, was colonized largely by European immigrants in the 19th century, and presently has a high proportion of Caucasians. However, people of Amerindian and African descent are also frequent in the population [21].

In the current study, a significant difference between the observed and expected heterozygosity was observed for *HLA-B*. Despite a high degree of heterozygosity (90.7%), the number of heterozygous individuals was lower than expected. This difference can be explained by the composition of our sample, i.e. non-healthy individuals. This deviation from Hardy-Weinberg proportions may also be related to the pathological condition itself, whether or not it is related to genetic causes; or because we did not exclude individuals who had some degree of kinship to each other [22, 23].

A total of 19 alleles of *MICA* were found in this study. Similar numbers of alleles were observed in Brazilian Caucasians [17], Afro-Americans and Euro-Americans [24], Moroccans [25] and in Murcia, Spain [20]. Although 19 *MICA* alleles were detected, a large number of these alleles were found with a frequency of only 1%. In addition, MICA*008, MICA*002, MICA*004 and MICA*009 together comprised more than 67% of the allelic distribution, and these alleles are also common in other populations [16, 17, 20, 24, 25].

The *MICA* allelic diversity found in this study is similar to the levels found by Marin et al. (2006) [16] and Ribas et al. (2008) [17] in samples from healthy Brazilian subjects. MICA*008 was the most frequent allele group, similar to findings in other Caucasian populations [5, 17, 20, 26, 27]. However, we also found a series of other alleles from different European, African and Asian populations that colonized the region [20, 28, 29].

As expected from the short distance between the *HLA-B* and *MICA* loci, a significant linkage disequilibrium was observed. The largest number of *MICA*–*HLA-B* haplotypes was found in Caucasian populations, such as MICA*009-B*51, MICA*004–B*44 and MICA*002–HLA-B*35 [7, 20, 26, 30–32]. However, these haplotypes were also found in Asian [18] and African population [29].

More typically, a single *MICA* allele is associated with several *HLA-B* alleles, whereas a few *HLA-B* alleles are associated with some *MICA* alleles [17]. In this study, the most common allele groups (MICA*008, -*002, and -*004) had several associations with *HLA-B*: MICA*008 was associated with HLA-B*15, -*07, -*44, -*08, -*13, -*40, -*37 and -*40; MICA*002 was associated with HLA-B*15, -*39, -*35, -*58 and -*43; and MICA*004 was associated with HLA-B*44, -*42, -*41, -*49 and -*48. In contrast, *HLA-B* alleles had associations with *MICA*, such as HLA-B*35 with MICA*002, MICA-*16, -*46 and *-52. In addition, most *HLA-B* alleles, such as the B*07 and *08 allele group, had only a single *MICA* association (MICA-*008).

This association may indicate a different evolutionary history of the *MICA* gene from classical *HLA*; the common alleles *MICA* are very old, predating major branches of the *HLA-B* alleles [24]. In vitro, the *MICA* allelic diversity may affect ligand binding between the MICA (strong or weak binders) and the NK-cell receptor NKG2D, affecting natural killer-cell activation and the modulation of T-cell responses [20, 24, 33, 34]. According to Gao et al. (2006) [24], future studies of the capacity of MICA to interact with NK-cell receptors across populations may provide information for population-based studies of diseases.

The frequencies of the MICA and HLA-B allele groups reported in this study were compared with those published by Ribas et al. [17], since we adopted the similar criteria used in that study. Our enrolled patients and the bone-marrow volunteer donors selected by Ribas et al. [17] came from the same geographic area (the same State) and showed the same pattern of ethnicity, predominantly Caucasians [21].

The present results are very similar to those found by Ribas et al. (2008) [17]. Among 23 haplotypes with a frequency above 1%, 20 had significant values of attraction in both studies. MICA*008-B*15 and MICA*009-B*35 did not show any significant values, so they are in linkage equilibrium. A discordant result was found for MICA*008-B*40, which showed a significant result only in the study by Ribas et al. (2008) [17]. MICA*002-B*39, MICA*002-B*38 and MICA*004-B*42 had D’ = 1 in both studies, making it possible to determine the *MICA* allele merely by knowing the *HLA-B* allele. Among the significant results, 7 haplotypes showed D' = 1 in the study of Ribas et al. (2008) [17], and D'<1 in our renal patients. These results suggest that despite the strong linkage disequilibrium observed in the majority of frequent haplotypes in our population, the linkage is not absolute, and as the sample size increases, the number of “D' = 1” values may decrease.

Another interesting result, as observed in the S1 Table, is the haplotype repulsion, indicating that 36 haplotypes that would be expected if the sample were in linkage equilibrium were not observed in this sample. Also, 8 haplotypes were found with significantly lower frequencies than expected. A very unusual combination expected for our population was found in haplotype MICA*045-B*47, the only combination that showed a correlation equal to 1 (r^2^ = 1, D’ = 1, *p* = 0.0000), i.e., a perfect correlation between two rare alleles (both 0.14%). This could be explained by inferring a recent in-migration of the family of the individual who provided the sample, with no direct association with the overall population. Although Ribas et al. (2008) [17] did not list samples with a frequency less than 1%, one can deduce that these values would not have appeared in that study, because the MICA*045 allele was not observed and the HLA-B*47 allele was observed, so the correlation is not absolute.

Comparison of *MICA–HLA-B* haplotype frequencies showed no significant difference between the haplotypes with a frequency above 1% in both studies. Notably, the MICA*027-B*40 and MICA*002-B*53 haplotypes, found in this study in frequencies of 3.32% and 2.02%, respectively, could be candidates for common haplotypes; however, we could not determine the exact frequencies that were observed in the healthy population.

Considering that several studies have suggested associations of the *HLA* haplotype with various diseases, the calculation of LD parameters was used for comparison with the results of health subjects also in the state of Paraná found by Ribas et al. (2008) [17], and not with the aim of determining the population structure.

In conclusion, the *MICA* allelic diversity in our population is similar to those of other Caucasian populations worldwide. However, we found a series of other allele groups, which may result from the contribution of alleles from different European, African or Amerindian populations that colonized the region. This study expands our knowledge of the distribution of *MICA* polymorphisms and linkage disequilibrium with *HLA-B* alleles, helping to elucidate possible associations with different diseases in patients with chronic kidney disease. Finally, our data could be useful as a preliminary clinical reference for better understanding of the mechanisms involved in the allograft rejection associated with *MICA* polymorphisms in the Brazilian population.

## Supporting information

S1 Table Graphic representation of the linkage disequilibrium between MICA and HLA-B alleles. The values shown in the fields are haplotype frequencies and in brackets the correlation index r2. 0% frequency was not represented. P values <0.05 were colored. Haplotypes in attraction with D' = 1 were colored in dark red. Haplotypes in attraction with D'<1 were colored in bright red. Haplotypes in repulsion with D' = -1 were colored in dark blue. Haplotypes in repulsion with D'>-1 were colored light blue.
(DOCX)
