# Supplementary material for: MICA diversity and linkage disequilibrium with HLA-B alleles in renal-transplant candidates in southern Brazil
Source: PLoS One. 2017 Apr 18;12(4):e0176072. doi: 10.1371/journal.pone.0176072 (PMC5395226; doi:10.1371/journal.pone.0176072)
Supplement: S1 Table — The values shown in the fields are haplotype frequencies and in brackets the correlation index r2. 0% frequency was not represented. P values <0.05 were colored. Haplotypes in attraction with D' = 1 were colored in dark red. Haplotypes in attraction with D'<1 were colored in bright red. Haplotypes in repulsion with D' = -1 were colored in dark blue. Haplotypes in repulsion with D'>-1 were colored light blue. (DOCX) [file pone.0176072.s001.docx]

Supplementary Table 1: Graphic representation of the Linkage Disequilibrium between *MICA* and *HLA-B* alleles.

| ***Locus*** |  |  | *MICA** | | | | | | | | | | | | | | | | | | |
| --- | --- | --- | --- | --- | --- | --- | --- | --- | --- | --- | --- | --- | --- | --- | --- | --- | --- | --- | --- | --- | --- |
|  | **Alelles** |  | *01* | *02* | *04* | *06* | *07* | *08* | *09* | *10* | *11* | *12* | *15* | *16* | *17* | *18* | *27* | *44* | *45* | *46* | *52* |
|  |  | **Freq.** | *1.30%* | *17.05%* | *14.88%* | *0.58%* | *2.46%* | *21.68%* | *13.73%* | *6.65%* | *3.03%* | *1.30%* | *1.01%* | *3.32%* | *2.02%* | *4.62%* | *5.64%* | *0.14%* | *0.14%* | *0.14%* | *0.29%* |
| HLA-B* | *07* | *6.50%* |  | (0.01) | 0.29% (0.01) |  |  | 5.49% (0.16) | (0.01) |  |  |  | 0.14% (0) |  |  |  | 0.58% (0) |  |  |  |  |
|  | *08* | *5.92%* |  | (0.01) | 0.14% (0.01) |  |  | 5.20% (0.16) | (0.01) |  |  |  |  |  |  |  | 0.58% (0) |  |  |  |  |
|  | *13* | *1.59%* |  | 0.14% (0) |  |  | 0.14% (0) | 1.30% (0.03) |  |  |  |  |  |  |  |  |  |  |  |  |  |
|  | *14* | *3.18%* | 0.14% (0) | (0.01) | 0.14% (0) |  |  | (0.01) |  |  | 2.89% (0.86) |  |  |  |  |  |  |  |  |  |  |
|  | *15* | *8.53%* |  | 0.14% (0.02) | (0.02) |  |  | 2.60% (0) | (0.01) | 5.20% (0.44) |  |  |  | 0.29% (0) |  |  | 0.29% (0) |  |  |  |  |
|  | *18* | *4.91%* | 1.16% (0.2) | (0.01) | (0.01) |  |  | (0.01) | (0.01) |  |  |  |  |  |  | 3.76% (0.6) |  |  |  |  |  |
|  | *27* | *3.03%* |  | (0.01) |  |  | 2.31% (0.71) | 0.29% (0) |  |  |  |  |  |  |  | 0.14% (0) | 0.29% (0) |  |  |  |  |
|  | *35* | *10.55%* |  | 5.64% (0.11) | (0.02) |  |  | 0.14% (0.03) | 1.45% (0) | (0.01) |  |  |  | 3.03% (0.24) |  | (0.01) | (0.01) |  |  | 0.14% (0.01) | 0.14% (0) |
|  | *37* | *1.30%* |  |  |  |  |  | 1.30% (0.05) |  |  |  |  |  |  |  |  |  |  |  |  |  |
|  | *38* | *3.32%* |  | 3.32% (0.17) | (0.01) |  |  | (0.01) |  |  |  |  |  |  |  |  |  |  |  |  |  |
|  | *39* | *3.47%* |  | 3.47% (0.17) | (0.01) |  |  | (0.01) | (0.01) |  |  |  |  |  |  |  |  |  |  |  |  |
|  | *40* | *5.20%* |  | 0.14% (0.01) | (0.01) |  |  | 1.59% (0) | (0.01) |  |  |  |  |  |  | 0.14% (0) | 3.32% (0.35) |  |  |  |  |
|  | *41* | *1.88%* |  |  | 1.73% (0.09) |  |  |  |  |  |  |  |  |  | 0.14% (0) |  |  |  |  |  |  |
|  | *42* | *2.17%* |  |  | 2.17% (0.13) |  |  | (0.01) |  |  |  |  |  |  |  |  |  |  |  |  |  |
|  | *44* | *9.97%* |  | (0.02) | 6.07% (0.18) |  |  | 3.18% (0.01) | 0.14% (0.01) | (0.01) |  |  |  |  |  |  | 0.58% (0) |  |  |  |  |
|  | *45* | *2.02%* |  |  |  |  |  | (0.01) | 1.16% (0.03) |  |  |  | 0.87% (0.36) |  |  |  |  |  |  |  |  |
|  | *46* | *0.14%* |  |  |  |  |  |  |  | 0.14% (0.02) |  |  |  |  |  |  |  |  |  |  |  |
|  | *47* | *0.14%* |  |  |  |  |  |  |  |  |  |  |  |  |  |  |  |  | 0.14% (1) |  |  |
|  | *48* | *0.43%* |  |  | 0.14% (0) |  |  | 0.29% (0.01) |  |  |  |  |  |  |  |  |  |  |  |  |  |
|  | *49* | *3.18%* |  | (0.01) | 3.03% (0.17) |  |  | (0.01) |  |  | 0.14% (0) |  |  |  |  |  |  |  |  |  |  |
|  | *50* | *2.46%* |  |  | 0.43% (0) |  |  | 0.14% (0) | 1.73% (0.07) | 0.14% (0) |  |  |  |  |  |  |  |  |  |  |  |
|  | *51* | *9.68%* |  | (0.02) | 0.29% (0.01) | 0.58% (0.05) |  | 0.14% (0.03) | 7.80% (0.4) | 0.58% (0) |  |  |  |  |  | 0.14% (0) | (0.01) |  |  |  | 0.14% (0.01) |
|  | *52* | *2.02%* |  |  |  |  |  | (0.01) | 1.45% (0.06) | 0.58% (0.02) |  |  |  |  |  |  |  |  |  |  |  |
|  | *53* | *2.17%* |  | 2.02% (0.09) |  |  |  | (0.01) |  |  |  |  |  |  |  | 0.14% (0) |  |  |  |  |  |
|  | *54* | *0.29%* |  |  |  |  |  |  |  |  |  | 0.29% (0.22) |  |  |  |  |  |  |  |  |  |
|  | *55* | *1.01%* |  |  | 0.14% (0) |  |  |  |  |  |  | 0.87% (0.57) |  |  |  |  |  |  |  |  |  |
|  | *56* | *0.14%* |  |  |  |  |  |  |  |  |  | 0.14% (0.11) |  |  |  |  |  |  |  |  |  |
|  | *57* | *2.60%* |  | 0.14% (0) | 0.29% (0) |  |  | (0.01) |  |  |  |  |  |  | 1.88% (0.66) | 0.29% (0) |  |  |  |  |  |
|  | *58* | *2.17%* |  | 2.02% (0.09) |  |  |  | (0.01) |  |  |  |  |  |  |  |  |  | 0.14% (0.07) |  |  |  |

The values ​​shown in the fields are haplotype frequencies and in brackets the correlation index r^2^.
0 % frequency was not represented. P values ​​<0.05 were colored. Haplotypes in attraction with D'=1 were colored in dark red. Haplotypes in attraction with D'<1 were colored in bright red. Haplotypes in repulsion with D'=-1 were colored in dark blue. Haplotypes in repulsion with D'>-1 were colored light blue.
